# A field study of the survival and dispersal pattern of *Lutzomyia longipalpis* in an endemic area of visceral leishmaniasis in Brazil

**DOI:** 10.1371/journal.pntd.0006333

**Published:** 2018-04-02

**Authors:** Fredy Galvis-Ovallos, Claudio Casanova, Denise Pimentel Bergamaschi, Eunice Aparecida Bianchi Galati

**Affiliations:** 1 Departamento de Epidemiologia, Faculdade de Saúde Pública, Universidade de São Paulo, São Paulo, Brasil; 2 Laboratório de Parasitoses por flagelados, Superintendência de Controle de Endemias, Secretaria de Estado da Saúde, Mogi Guaçu, São Paulo, Brasil; National Institutes of Health, UNITED STATES

## Abstract

Zoonotic Visceral leishmaniasis (ZVL) is a neglected tropical disease that in the Americas is caused by the infection of *Leishmania infantum* and the domestic dog (*Canis familiaris*) is the main parasite reservoir in urban areas. The parasite is mainly transmitted by populations of the sibling species *Lutzomyia longipalpis* that has been spreading in countries including Brazil, Argentina, Paraguay and more recently Uruguay. Although bionomic parameters such as population survival and the duration of the gonotrophic cycle are critical in evaluating vector capacity, field studies have rarely been applied to sand fly populations. The present study sought to evaluate basic bionomic parameters related to the vectorial capacity of the (S)-9-methylgermacrene-B population of the *Lu*. *longipalpis* complex in a visceral leishmaniasis area of Sao Paulo state. The daily survival rate, the duration of the gonotrophic cycle and the dispersal pattern were evaluated through the mark- release-recapture method. A total of 1,547 males and 401 females were marked and released in five experiments carried out between February 2013 and February 2014. The higher recapture rates occurred within 100 meters of the release point and the estimated daily survival rates varied between 0.69 and 0.89 for females and between 0.69 and 0.79 for males. The minimum duration of the gonotrophic cycle observed was five days. The absolute population size, calculated ranged from 900 to 4,857 females and from 2,882 to 9,543 males. Our results demonstrate a high survival rate of this vector population and low dispersal that could be associated with the presence of all necessary conditions for its establishment and maintenance in the peridomiciles of this area. Our findings contribute to the basic data necessary for the understanding of ZVL dynamics and the evaluation of the implementation of prevention and control measures.

## Author summary

American Visceral leishmaniasis (VL) is an anthropozoonosis resulting from infection by *Leishmania infantum* mainly transmitted by the bite of females of the sand fly *Lutzomyia longipalpis* which feed mainly on domestic dogs. It is a serious tropical disease that in some countries has spread in association with several factors such as human migration, precarious urbanization and vector adaptation to urban areas. In Brazil, although control measures such as the elimination of infected dogs, the application of residual insecticide and environmental management have been applied by the health authorities, however, they have not resulted in any significant reduction in VL incidence. The knowledge of bionomic parameters of vector populations is critical for the understanding of the transmission dynamic of VL and to evaluate prevention and control measures. Therefore, in the present study we applied the mark-release-recapture (MRR) method to evaluate under field conditions some basic bionomic parameters of a population of the *Lutzomyia longipalpis* complex. Our findings demonstrate a high survival rate of this vector and a low population dispersal. These results suggest that measures applied to dog populations to prevent sand fly bites could contribute to the reduction of the surviving vector population and indirectly to the reduction of the host biting rate. Additionally, the low dispersal suggests that environmental management could contribute to the reduction of vector density. Our data serve as the first estimates of vector survival rates of populations of the sibling species *Lu*. *longipalpis* under natural conditions and could be applied in the evaluation of prevention and control measures.

## Introduction

Visceral leishmaniasis is a neglected tropical disease (NTD) that presents a complex dynamic influenced by socioeconomic factors such as poverty and limited access to health services [1,2] and also specific biological factors related to vector and host ecology parameters as well as natural interactions between etiological agents, hosts and vectors [3,4]. Although in recent years some initiatives have been undertaken with a view to the control of VL [2], some gaps in our knowledge of VL transmission dynamics could hinder these efforts [5]. In Latin America the need to clarify some key aspects of leishmaniasis transmission [6] and the failure of control programs together with the emergence of VL in new geographical areas in South America [7,8] stress the importance of this observation.

VL in the Americas is caused by *Leishmania infantum* and populations of the *Lu*. *longipalpis* complex are undoubtedly its main vector, although in the last years some sand fly species has been pointed as permissive vectors of this agent [9, 10, 11, 12]. The domestic dogs constitutes the main reservoir in urban areas [13,14,15]. In Brazil between 1999 and 2014, 53,067 human cases were notified [16] and currently represent around 95% of the cases notified in Latin America. Though the Brazilian northeast region is the most affected, recently VL has emerged in several areas of the Southeast region, an event associated mainly with the dispersion of populations of the *Lu*. *longipalpis* sibling species [17,18]. Ecological differences in these populations could be associated with different epidemiological patterns of VL transmission as suggested by Casanova et al [17] in relation to Sao Paulo State.

Beside the prevalence of host infection and the presence of the susceptible population, the maintenance of transmission cycles is dependent on ecological parameters that act directly on vector capacity [19,20] and therefore their estimation permits the evaluation of the role of sand fly species in VL transmission [4, 21, 22]. Among vector parameters, population size and the duration of the gonotrophic cycle are indirect indicators of the frequency and intensity of the host biting rate [23, 24, 25], while the vector life expectancy indicates the infective life and the proportion of the population capable of transmitting an agent [23, 25, 26]. Thus, these estimates have important applications in epidemiological surveillance and contributes to the understanding of VL dynamics [27, 28, 29].

Although these bionomic parameters can be evaluated under field conditions by means of the mark-release-recapture method (MRR), commonly used in malaria and arbovirus vector studies [19, 23], this method has rarely been applied to evaluate ecological parameters of neotropical sand fly populations [30,31,32,33] Interestingly, the knowledge of sand fly dispersal permits the analysis of the applicability of control programs such as those of environmental management and the application of residual insecticides. With the recognition that *Lu*. *longipalpis* constitutes a complex of cryptic species [34,35,], many gaps in vector ecology have come to light which hinder the understanding of zoonotic visceral leishmaniasis (ZVL) dynamics in Latin America.

In the present study, we have set out observations on the dispersal, population size, duration of gonotrophic cycle and estimated survival of a population of the *Lu*. *longipalpis* complex (S)-9-methylgermacrene-B) and analyzed the results in the light of their significance in relation to visceral leishmaniasis dynamics in a highly endemic area of VL in Brazil.

## Methods

### Study area

The study was undertaken in the urban area of Panorama municipality, located in the western region of São Paulo state, Brazil. This locality belongs to the Atlantic forest region and presents a tropical climate **Aw** according to Koppen's classification [36]. Temperatures vary between 12 and 35°C with a rainy season occurring between October and March. The predominant soil is sand with flat topography. In 2012, this municipality was considered an area with intense transmission of ZVL according to the visceral leishmaniasis program classification of the health secretariate of Sao Paulo state. From 2013 to 2015, due to the decrease in the number of reported cases, this municipality was classified as an area of sporadic transmission. Previous study in this locality had identified six sand fly species belonging to the *Nyssomyia*, *Evandromyia*, *Brumptomyia* and *Lutzomyia* genera, with the predominance of the (S)-9-methylgermacrene-B population of the *Lu*. *longipalpis* complex [18].

### Mark-release-recapture method

Five experiments were undertaken between 2013 (February, April, September, October) and 2014 (February). Two experiments were undertaken in a domicile located in the "Bela Vista" neighborhood (21°22'08''S, 51°51'12''W) and three other experiments in a domicile located in the "Areia Branca" neighborhood (21°21'47''S, 51°50'36W) in Panorama municipality. The distance between those domiciles is around 1.3 km.

The domiciles were selected due to the high frequencies of *Lu*. *longipalpis* and presence of favorable conditions (vegetation near the domiciles and animal husbandry) for the captures of this species. The residents of these domiciles provided formal consent to undertake the sand flies captures. The captures were performed with a Castro aspirator [37] in the animal shelters (chicken houses and pigsty) between 18:00 and 22:00 hours. This method was used to increase the chances of capturing naturally engorged females to estimate the duration of the gonotrophic cycle. All females were captured after a natural and full blood feeding on chicken or pigs. In each experiment, the specimens captured were sorted out by sex and subsequently marked with fluorescent powder (BioQuip) in color pink using a device described by Casanova et al. [31] and released after 24 hours. In the February (2013) experiment the sand flies marked were released at a distance of 60 m from the capture point to evaluate their dispersal and loyalty behavior. In the other four experiments the specimens were released at the capture point (0 meters).

### Recapture method

The recapture attempts began within 24–72 hours after the release and were performed on 6–14 consecutive days. Detailed information (capture effort, temperature, distance of the release point from the CDC traps, temperature) for each experiment is presented in Table 1.

**Table 1:** General information related to the mark-release-recapture experiments and estimates of *Lutzomyia longipalpis* density, Panorama, São Paulo state, Brazil.

	Feb/13	Apr/13	Sep/13	Oct/13	Feb/14
Release data	18/02	25/04	24/09	26/10	27/02
Beginning of recaptures	21/02	27/ 04	27/09	29/10	01/03
Mean maximum temperature (°C)	34.0	31.3	27.0	27.1	32.4
Precipitation (mm)	1.9	0	66.9	41	120
Days of recapture attempts	3–17	2–16	1–10	1–10	1–10
Distance between the release and capture points (m)	60	0	0	0	0

**Fig 1:**
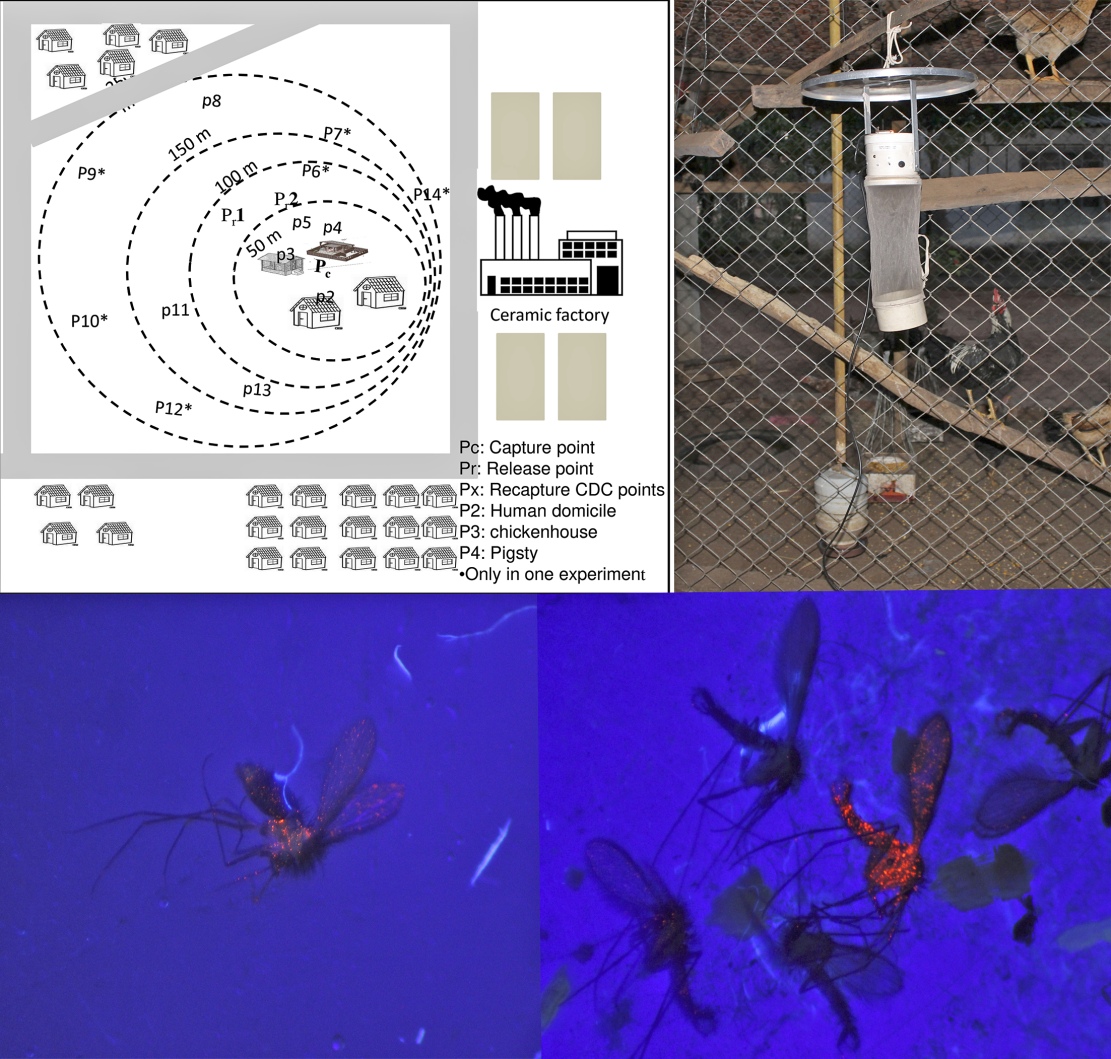
Schematic diagram of the distribution of the recapture points in the recapture attempts and marked and unmarked specimens.

The recaptures were performed with a Castro aspirator from 19:00 to 22:00, on five consecutive days, on the walls of the domestic animal shelters and with 6–13 CDC light traps installed between 18:00 and 07:00 hours within 250 meters of the release point (Fig 1). All the specimens collected in each recapture attempts were euthanized (in freezer) and observed in the laboratory under stereoscopy illuminated with UV light to identify the insects marked with fluorescent power. Thereafter all the specimens were clarified and morphologically identified [38].

### Gonotrophic cycle estimation

During three experiments (Feb-2013, Apr-2013 and Feb-2014) engorged females were captured in chicken houses, counted, marked and released 24 hours later. The recaptures were performed as described above. All the recaptured females were immobilized by cooling during 3 minutes at 4°C and observed under UV light, and those marked were individualized in vials containing a moist plaster layer at the bottom where the females laid their eggs or were dissected to observe their parity status. The duration of the gonotrophic cycle was considered to be the time between the blood meal (day of capture) and the median time of oviposition, in view of the fact that this *Lu*. *longipalpis* s.l population presents gonotrophic concordance as observed in previous studies [39].

### Statistical analysis

Comparisons of the frequencies of males and females by capture method (manual captures vs CDC light traps) and distance were performed using the Chi-squared test and the probability and odds of capture by the distance from the release point were calculated.

The mortality was considered constant during the period of recapture. Thus, the daily survival was estimated horizontally on the basis of the numbers of specimens recaptured, which were transformed by the ln (y+1). A linear regression analysis was performed as a function of the delay (days) in recapture after the release [25, 26]. The daily survival estimate was obtained by the equation ln *mt—*ln (*M*_*0*_*r*)–*t* ln*s*, where *M*_*0*_ is the number of specimens marked and released, *r* is the proportion of specimens recaptured, *t* is the period (days) of recapture attempts. Thus, the daily survival *s* is obtained from the antilog of the slope obtained in the regression analyses. The daily survival rate was estimated by the expression *s* = *e*^*b*^, where e is the natural logarithm base and b is the regression coefficient [26, 32, 40].

The Lincoln index, as corrected by Bailey [41], was used to estimate the population size (*N*). Using this deterministic method, the population size is estimated using a unique mark event. This estimation was undertaken using the equation *N* = *a*(*n*+1)/(*r*+1); where *a* is the number of mark-release specimens, *n* is the number of specimens recaptured, and *r* the number of marked specimens recaptured. The variance of N was estimated according to the equation *a*^2^*n* (*n*-*r*)/[(*r*+1)^2^ +(*r*+2)]. The confidence interval was estimated using 1.96 standard deviations (±1.96). The male and female density/km^2^ was estimated of the population size divided by the area covered by the CDC traps, 0.25 km^2^ [42].

## Results

### Description of the recapture data

In the five experiments a total of 1,547 males and 401 females were marked and released. During the recapture attempts 4,780 males and 1,096 females were captured and a total of 242 males and 36 females were recaptured, giving recapture rates of 15.64% and 8.98%, respectively. The number of males and females capture by method and distance from the point of origin is presented in Table 2.

**Table 2:** Number of females and males of *Lutzomyia longipalpis* captured and recaptured by method and distance from the release point during the mark release recapture experiments.

Capture method	Distance from release point	Capture effort (hours)	Females captured	%	Females recaptured	%	Males captured	%	Males recaptured	%
CDC traps	0–50	2760	277	25.3	3	8.3	994	20.8	25	10.3
	51–100	1764	255	23.3	9	25.0	888	18.6	25	10.3
	101–150	1140	30	2.7	0	0.0	125	2.6	0	0.0
	151–250	636	4	0.4	0	0.0	16	0.3	2	0.8
	Subtotal	6300	566	51.6	12	33.3	2013	42.3	52	21.5
Manual captures	0–100	72	530	48.4	24	66.7	2757	57.7	190	78.5
Total		6372	1096	100	36	100	4780	100	242	100

Considering all the specimens captured in the recapture attempts a statistical association between the methods of capture and the sex of species was observed and showed more females captured by the manual method (p<0.001).

### Population dispersal

During the recapture attempts 97% of males and females (marked and unmarked) were captured within a distance of 100 m from the release point. No female was recaptured further than 100 meters and only two males were recaptured between 150 and 250 meters from the release point (Table 2). The proportion of captures was different according to the distance from the release point (p<0.001) with a greater chance of capture within 50 meters (odds ratio of 13.6) and decreasing with distance from the release point. In the first experiment in which the specimens were released at a distance of 60 m from the original capture point, 98% of the specimens recaptured were collected in the animal shelters where they had initially been captured.

As regards the captures with CDC light traps, a predominance of males (3:1) was observed and this proportion was no different in terms of the distance from the point of release in the two areas where the experiments were performed (domiciles 1 p = 0.717; domicile 2 p = 0.524).

### Population survival and size

The daily survival rate for females was estimated during three experiments (month of high frequencies) and the values ranged from 0.79 to 0.89. In the regression analysis the highest coefficient of determination (0.63) was obtained in the February/2013 experiments. For the males, estimates of daily survival were obtained from the five experiments and the values ranged from 0.69 to 0.74. We considered a male daily survival rate of 0.74, a value estimated in the April/2013 experiment since it presented the highest coefficient of determination as representative of male survival (Table 3).

**Table 3:** Estimates of daily survival for females and males of *Lutzomyia longipalpis* estimated horizontally from the decrease in the numbers of insects recaptured, transformed by ln (*y* +1) and subject to regression analysis as a function of time (days) since release.

Month/year	Females				Males			
	b	R^2^	*P*	s	b	R^2^	*P*	s
February/13	-0.23	0.63	0.006	0.79	-0.29	0.67	0.000	0.74
April/13	-0.12	0.35	0.012	0.88	-0.29	0.81	0.000	0.74
September/13	-	-	-	-	-0,369	0.69	0.003	0.69
October/13	-	-	-	-	-0.236	0.36	0.030	0.79
February/14	-0.11	0.33	0.077	0.89	-0.34	0.72	0.001	0.71

b:regression coefficient;R^2^: determination coefficient; P: p-value, s: survival rate

**Fig 2:**
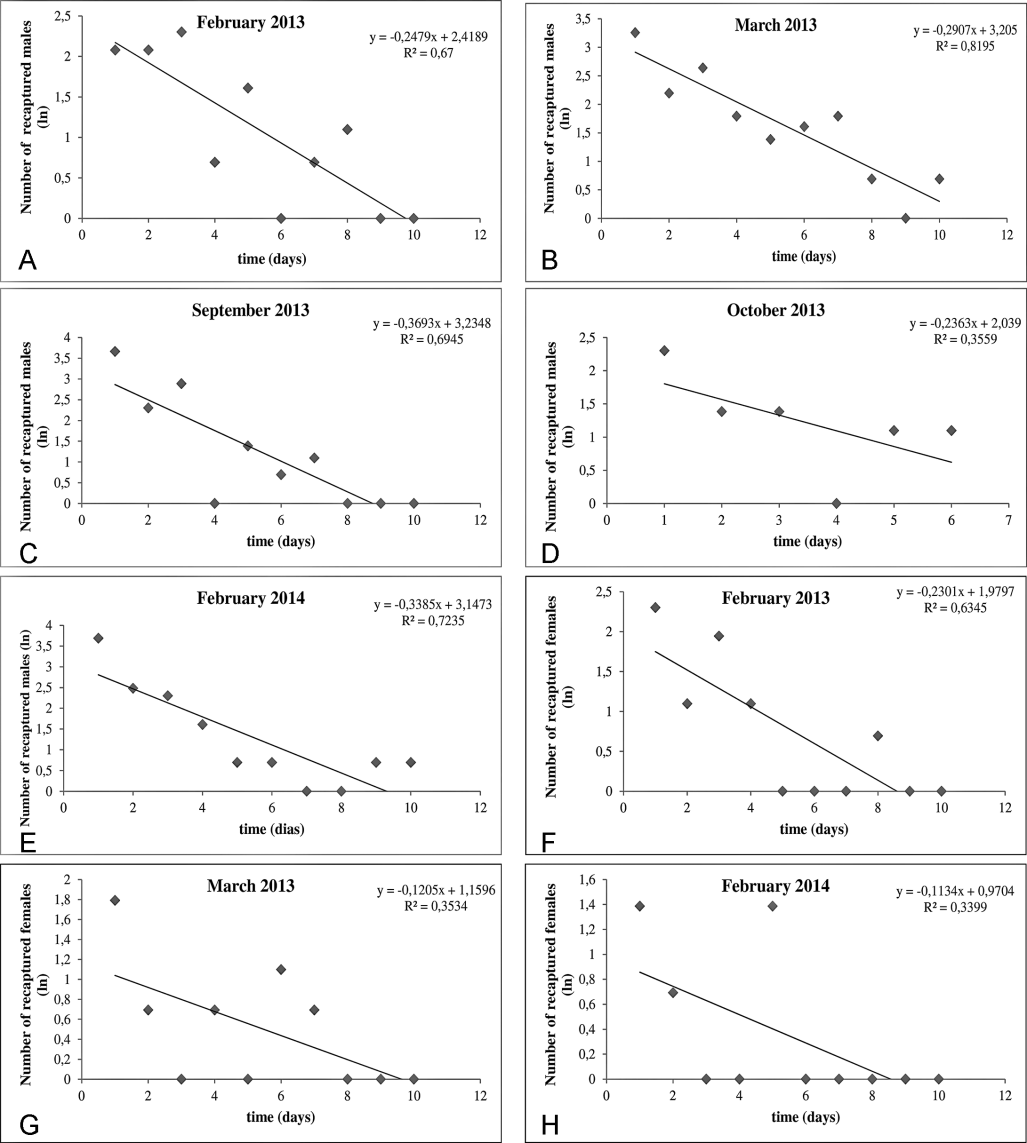
Scatter plot illustrating the relationship between the number of recaptured specimens and the time of recapture attempts in each experiment for males (A-E) and females (F-H).

The scatter plots of the regression analysis of the number of males and females recaptured during the recapture attempts are presented in Fig 2.

The female population size calculated by the Lincoln Index ranged from 900 (September/2013) to 4,857 females (February/2014). For the males, the population size ranged from 2,882 (September/2013) to 9,543 males (February/2014). The density per km^2^ of females ranged between 3,600 and 19,426 and for males between11,528 and 38,172. A larger population of males than females was observed in all the experiments (Table 4).

**Table 4:** Estimates of population parameters for females and males of *Lutzomyia longipalpis* in Panorama municipality, São Paulo state, Brazil.

Females								Males						
Month/year	a	r	N	PS	(SD)	CI	Density/Km^2^	a	r	n	PS	(SD)	CI	Density/Km^2^
February/13	174	19	286	2648	529	1610–3685	10,591	390	30	733	9543	1597	6412–12674	38,172
April/13	70	9	404	3151	844	1496–4805	12,604	310	62	1678	8396	1013	6410–10382	33,584
September/13	29	3	91	900	295	322–1478	3,600	332	59	511	2882	340	2214–3550	11,528
October/13	45	1	100	4546	1299	2000–7091	18,184	200	20	548	5491	1093	3348–7634	21,964
February/14	83	4	233	4857	1568	1782–7931	19,426	315	60	1494	7850	960	5968–9732	31,399

**a** number of specimens marked and released

**r** number of specimens recaptured

**n** total number of specimens captured during the recapture attempts

**PS** population size

**SD** standard deviation

**CI** confidence interval

### Duration of gonotrophic cycle (GC)

As regards the duration of the gonotrophic cycle, in the February/2013 experiment: of the 174 engorged females marked and released, two were recaptured without either eggs or blood in their abdomen on the fifth day after their release. The temperature during the experiment ranged between 21 and 33°C and a minimum of 5 days was estimated for the duration of the GC. In the September/2013 experiment the temperature ranged between 17 and 32°C and one female was recaptured four days after its release. This female laid eggs on the sixth day, suggesting a minimum GC of 6 days.

## Discussion

Knowledge of vector population parameters is fundamental to the understanding of the transmission dynamics of vector-borne diseases such as VL [42]. Although sand fly population survival rates influence other characteristics of vector capacity, such as population size and infective life time [23, 25], few studies have been undertaken under natural conditions to estimate this parameter [26, 43]. For neotropical sandflies some information has been obtained for vectors of cutaneous leishmaniasis by MRR [26,32], therefore this method has been used to estimate population dispersal in VL vectors [43]. In the present study we estimate using MRR the ecological parameters of one of the sibling species of *Lu*. *longipalpis* that occur in Sao Paulo state (S-9 methyl germacrene). We observed that recapture rates of females ranged from 2.2% to 12.8%, and for males from 7.7% to 20.7%. These results are similar to those described for other populations of *Lu*. *longipalpis* s.l in VL endemic areas in Colombia [44] and Brazil [45]. Lower recapture rates have been reported [33] in Campo Grande-MS (Brazil), however this result may have been affected by the long interval of the recapture attempts.

Regarding population dispersal, in the five experiments the majority of the specimens were recaptured at distances of less than 100 meters from the release point, contrasting with the observations made in Colombia that described high recapture ratios at distances of between 100 and 300 m and dispersion of almost 500 meters [44]. This difference could be explained by the distinct environmental characteristics of the areas studied, since in the Colombian rural areas researched, the blood sources were more widely dispersed and there was denser vegetation cover with more potential resting sites for sandflies. Corroborating our results, the limited dispersion of *Lu*. *longipalpis* populations has been described in other endemic VL areas in Brazil [31,43], as well as that for other sand fly species [30,31,32,46, 47].

The high male recapture rates and limited dispersion could be related to the characteristic aggregation (leks) of *Lu*. *longipalpis* s.l. [45] and the presence in the peridomicile of the basic resources (organic matter, humidity, protection from sunlight) favoring the development of immature forms, as well as of resting sites and blood feeding sources for adults, such as have been suggested for other sand fly species [30,31,32,46]. Thus, our results could suggest that environmental characteristic such as topography, vegetation cover and urbanization could also determine the natural population dispersal, since studies have demonstrated that this species can travel distances of up to 960 meters in two days [44]. Further to the dispersal observations, in the first experiment when the specimens marked were released at 60 meters from the original capture point, it was observed that they returned to the animal shelters where they had originally been captured. This observation suggests that the insects are able to memorize their area of activity and that there is an olfactory memory or loyalty mediated by the kairomones emitted by the host, corroborating previous observations for this species [49]. This behavior does not seem to be exclusive to this sand fly since similar observations have been made for *Ny*. *neivai* [47] and *Ny*. *whitmani* [48].

On the other hand, we estimate a daily survival rate ranging between 0.79 and 0.89 for females and 0.69 and 0.79 for males. These estimates could be understated due to uncontrolled factors such as emigration, deaths and recapture success [25,26]. However, these results constitute the first published data for populations of *Lu*. *longipalpis* under natural conditions. Our results suggest a high daily survival of the males and females of this species as compared to those described for *Ny*. *neivai* (0.64) and *Ny*. *intermedia* (0.57), vectors of tegumentary leishmaniasis in Brazil [26,32]. The survival estimates for the female population is difficult due to their low density in urban areas, as has been observed in previous studies [44]. The low recapture rate for females observed in the present study could be related to the fact that the females were engorged when released. This fact would influence their recapture rate since they could stay in their natural shelters until oviposition, making their capture more difficult.

In the first experiment a male was captured on the 13th day after release and a female on the 11th day, these periods being respectively the maximum recapture times observed. These values are in accordance with the recapture time described for *Lu*. *longipalpis* of 8 days [44]. However, dispersal studies have shown that males of this species can be recaptured even more than a month after release [33].

The estimated population size presents the lowest value in September/2013, with 2,883 males and 667 females. The maximum values were obtained in February/2013 with an estimated 9,234 males and 2,497 females. The greater male population seems to be related to their high level of activity of males in the animal shelters and also to the aggregation behavior (lek) observed for this species [45]. The estimated population size shows the high frequencies of *Lu*. *longipalpis* in this municipality in the rainy season as observed in a study with monthly captures in this focus [18] where females carrying eggs were also captured in the intradomicile. This information suggests that in this period the probability of contact between competent vector and infected and susceptible host increased. Therefore, the high survival of females together with the population size observed in the present study support the hypothesis that in the rainy season the risk of transmission of *Leishmania infantum* to the human and canine population in this locality increases.

The estimates of the duration of the gonotrophic cycle should be interpreted with care in view of the low frequencies of females captured and the low recapture rate. Although an alternative manner to evaluate this parameter under natural conditions would be to release engorged females reared in the laboratory as described by Morrison et al. [44], this practice is impossible in urban areas because of the ethical implications. An alternative method is the capture of naturally engorged females in animal shelters or the offer of a blood meal by the exposure of a host. Using this methodology, Dye et al. [45] released engorged females of *Lu*. *longipalpis* s.l and recaptured some of them without blood or eggs three days later. That suggests this period as being the minimum GC for this species in an Amazonian area. In the present study a minimum time ranging between five and six days for the GC was observed, a variation affected mainly by the temperature variation during the experiments (Table 1), however, other variables such as the physiological age must be considered. Under laboratory conditions a minimum GC duration of four days, with a median of five days, has been observed [39]. For *Phlebotomus papatasi*, under natural conditions, females take a blood meal every six days [50] and for the neotropical species *Ny*. *neivai*, Casanova et al [31] estimated a GC of four days.

### Epidemiological importance of the findings

Our observations demonstrated the long daily survival of one population of the *Lu*. *longipalpis* complex under field conditions, for the first time. Considering a daily survival of 0.79 for *Lu*. *longipalpis* (Table 3) and a minimum extrinsic incubation period of 7 days for *Le*. *infantum* [51], our data suggest that after a first infective blood meal on a competent reservoir, at least 19% of the potentially infective females will be able to take a second blood meal. According to our estimates of the size of the female population, it is to be expected that in a population of 4,857 females (Table 3), 923 females will still be alive on the fifth day. Thus, in this case, the infective population of the vector will depend on some other ecological parameter such as the biting rate or the prevalence and movement of a competent reservoir [19,20].

In view of the high vector survival rate observed, the use of control measures aiming to impact the survival of the vector population (e.g. the use of residual insecticides and canine host protection measures such as insecticide impregnated collars or topical products) could impact *Le*. *infantum* transmission in endemic areas. This reduction in the infective life of the vector population and the decrease in the host biting rate have been demonstrated both in field studies and mathematical models to affect the transmission dynamic [27,52].

We also have presented evidence that supports the hypothesis of loyalty behavior by the recapture of specimens in the animal shelters of previous nocturnal activity and the low population dispersal associated with conditions favorable to its development. This information supports the idea that measures such as environmental management in the peridomiciliary environment could impact the vector density as has been demonstrated in some endemic areas [53,54]. Because it seems that in these peridomiciles the sand fly population find all the elements necessary for their development (breeding sites, natural shelters and blood feeding sources) [12]; therefore, with the modification of these conditions the pathogen’s transmission can be reduced.

With the current expansion of VL to new areas in Brazil, estimates of vector population parameters are critical for the assessment of the vector capacity of the populations of this cryptic species, information necessary for the development of models to evaluate prevention and control measures as well as to increase our knowledge of VL dynamics [5].

## Conclusion

In conclusion, the S-9 *methyl* population of *Lu*. *longipalpis* has a long daily survival rate that can influence other parameters of its vectorial capacity such as infective life expectancy and the host biting rate. Its low dispersal seems to be associated with loyalty behavior and the presence of all the necessary conditions for the establishment and maintenance of the vector population. Our findings related to the population’s daily survival rate provide the first published information on Neotropical species involved in the transmission of the agent of VL in the Americas. Some prevention and control measures aiming at the decrease of vector density, such as environmental management and reduction of the vector survival rate by the use of insecticide impregnated collars or topical products on the canine population are strongly to be recommended. Our observation is of great value in the evaluation of control measures and in the development of mathematical models of VL dynamics.
